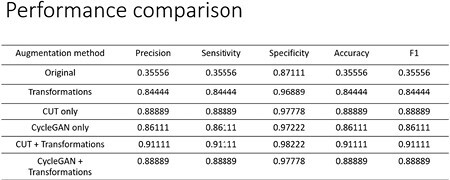# 39 Deep Learning and Mechanical Ventilation Period Based Inhalational Injury Score

**DOI:** 10.1093/jbcr/irae036.039

**Published:** 2024-04-17

**Authors:** Alan Pang, Yifan Li, Jowoon Chong

**Affiliations:** Texas Tech University Health Sciences Center, Lubbock, TX; Texas Tech University, Lubbock, Texas; Texas Tech University Health Sciences Center, Lubbock, TX; Texas Tech University, Lubbock, Texas; Texas Tech University Health Sciences Center, Lubbock, TX; Texas Tech University, Lubbock, Texas

## Abstract

**Introduction:**

Smoke inhalation is a serious complication associated with burn injuries. It notably increases mortality rates and prolongs hospital stays due to the thermal, chemical, and metabolic injuries inflicted on the airways by smoke. The current mainstream grading system, the Abbreviated Injury Score (AIS), a macroscopic evaluation of a microscopic disease process, has shown inconsistencies in accurately diagnosing and grading inhalation injury severity, largely due to its subjective nature. Our study aims to bridge these gaps by utilizing deep learning and artificial intelligence, proposing a novel method utilizing a Convolutional Neural Network (CNN) for more objective grading. Given the difficulty in collecting bronchoscopy images, we employed Contrastive Unpaired Translation (CUT) for data augmentation and conducted statistical analysis on lab work to pinpoint features impacting inhalation injury severity.

**Methods:**

We categorize the inhalation injuries into 6 degrees based on ventilator days: 1) under 24 hours as degree 1, 2) 1-2 days as degree 2, 3) 3-7 days as degree 3, 4) 8-14 days as degree 4, 5) 15-30 days as degree 5, and 6) after 30 days as degree 6. To augment the dataset volume, graphic transformations, and Contrastive Unpaired Translation (CUT) were utilized. The augmented dataset was then evaluated using GoogLeNet, and a Random Forest algorithm was implemented to assess feature importance meticulously.

**Results:**

Graphic transformations increased the image dataset from 153 to 1377 images, while Contrastive Unpaired Translation (CUT) further doubled the dataset to 2754 images. For evaluation, 30% of the original images were designated as the testing set and subjected to classification analysis via GoogLeNet. The enhancements through transformations and CUT propelled the classification accuracy from 36% (on original images) to a significant 91%. Statistical analysis highlighted that Total Body Surface Area (TBSA), Initial PH, Initial PaCO2, Pressors, Carboxyhemoglobin Level, and Resuscitation Fluid Amount Needed during the first 24 hours are pivotal determinants in assessing the severity of inhalation injuries.

**Conclusions:**

This study underscores the potential of leveraging advanced computational techniques to ameliorate the accuracy of inhalation injury grading in burn victims. Through graphic transformations and Contrastive Unpaired Translation (CUT), a significant augmentation in the image dataset was achieved, facilitating a more robust analysis. The proposed methodology not only elevates the precision in grading inhalation injuries but also initiates a path towards more objective evaluations in clinical settings, thus aiding in better-informed medical interventions for burn victims.

**Applicability of Research to Practice:**

If we can develop an objective measurement of inhalational injury with prognostic ability, then we can develop novel therapeutics and objectively measure their efficacy in treatment.